# An analysis of National Cancer Institute-funded scale-up research

**DOI:** 10.3389/frhs.2025.1624733

**Published:** 2025-09-19

**Authors:** Cynthia Vinson, Aubrey Villalobos, Margarita Correa-Mendez, Gila Neta

**Affiliations:** ^1^ Division of Cancer Control and Population Sciences, National Cancer Institute, National Institutes of Health, Rockville, MD, United States; ^2^ Center for Global Health, National Cancer Institute, National Institutes of Health, Rockville, MD, United States

**Keywords:** scale-up, cancer control, research gaps, evidence-based innovations, implementation science

## Abstract

**Background:**

The National Cancer Institute seeks to support cancer research to advance scientific knowledge that will “help all people live longer, healthier lives.” To do this, we need to understand how to effectively and efficiently scale-up evidence-based cancer control innovations (EBIs). We analyzed National Cancer Institute (NCI)-funded implementation science (IS) grants to understand gaps and opportunities for scale-up research.

**Methods:**

The National Institutes of Health (NIH) Query, View, and Report (QVR) system was used to identify NCI-funded IS grants focused on scale-up since 2016. Key search terms were identified, and two coders reviewed specific aims to identify IS and scale-up grants. Eligible grants were coded for study characteristics, including administrative, cancer-related, and scale-up related features using Microsoft Excel and iSearch.

**Results:**

Of the 61 grants initially identified, 17 were included. Approximately one-third of the grants were conducted abroad (*n* = 6). Most examined factors related to scale-up (e.g., barriers/facilitators, context) (*n* = 11). Nine studies assessed the costs and benefits of the scaled-up delivery of an EBI, and seven studies evaluated an implementation strategy for EBI scale-up. Most focused on prevention (*n* = 11), with seven focusing on screening. Cervical cancer (*n* = 6) was the most frequently studied cancer type. Most of the research took place in healthcare settings (*n* = 11).

**Conclusions:**

The NCI has funded a limited number of IS grants focused on scale-up. This analysis helps identify the current scope of the NCI portfolio and enables exploration of gaps and opportunities for future research on scale-up across the cancer continuum.

## Background

The National Cancer Institute (NCI) seeks to support cancer research to advance scientific knowledge that will “help all people live longer, healthier lives” (1). To do this, we need to understand how to effectively and efficiently implement evidence-based cancer control innovations (EBIs) at scale, across all settings and for all populations who could benefit. However, the scope of individual grant-funded research studies is often small-scale, in a single setting or delivery location with a limited number of study participants. Intervention research often asks, “what is the effect of this innovation in a given population and setting?”, and implementation research might ask “how can specific strategies improve the adoption of an EBI in a given population and setting?” Scale-up research then focuses on examining how to bring an EBI to multiple sites or settings and reach a greater swath of the population.

Research focused on scaling up EBIs is very limited to date, but an emerging priority with particular traction in low- and middle-income countries (LMICs). One review reported fourteen scale-up studies between 2003 and 2016, none of which focused on cancer, and another reported twenty-seven between 2010 and 2019 (2, 3), only one of which addressed cancer control (e.g., cervical cancer screening). Much of the research has focused on examining factors that influence scale-up, such as context and other barriers and facilitators, with less focus on testing strategies for scale-up. Greenhalgh and Papoutsi (2019) distinguish between the spread of an EBI, which they liken to replication in a new location, and scale-up, which they describe as “building infrastructure to support full scale implementation,” noting that both spread and scale-up have proven challenging (4). While the NCI has funded studies focused on the former concept of spread, replicating EBIs in new settings (e.g., testing strategies to optimize implementation of colorectal cancer screening programs in a Federally Qualified Health Center), very few have focused on scaling up EBIs. In their rapid review, Greenhalgh and Papoutsi discuss the promise of applying implementation science to the challenge of implementation at scale. The structured processes for the integration of EBIs in practice offered by implementation science theories, models, frameworks, and methodologies could be well-suited to advancing the widespread implementation of EBIs with local adaptation as needed to achieve positive health outcomes at scale (4). Thus, the NCI has supported implementation science to accelerate the use of evidence-based cancer prevention and screening strategies nationally (5) but has received and awarded very few studies focused on scaling strategies.

There is a desire to scale-up EBIs toward achieving the goal of all people living longer, healthier lives, and a recognition of the potential of implementation science to advance this work, yet the extent to which cancer control implementation science has focused on scale-up is unknown. Therefore, we analyzed NCI-funded implementation science grants to understand gaps and opportunities for scale-up research. The specific objectives were to understand the types of studies that have been funded to date and to characterize grants related to their implementation and cancer control focus, and scale-up related design features.

## Methods

### Study sample identification

Following established best practices in grant portfolio analyses (6, 7), this study used the NIH internal-use-only Query, View, and Report (QVR) system to identify cancer control implementation science grants focused on scale-up. The QVR search included a focused query using keywords within the title, abstract, and specific aims and specific filter criteria to identify the most relevant grants. To identify targeted keywords for our search, we referenced several review articles focused on scale-up and spread (2–4, 8) to identify key terms for scale-up research, and we used key terms for implementation science based on previously published implementation science portfolio analyses (6, 7). The search terms included “improvement science” OR “improvement research” OR “implementation science” OR “implementation research” OR “implementation framework” OR “healthcare delivery” OR “cancer care delivery” and at least one of the following terms: “scale-up” OR “scale up” OR “scaling up” OR “scaling out” OR “scaling-up” OR “scalability” OR “spread.”

**Figure 1:**
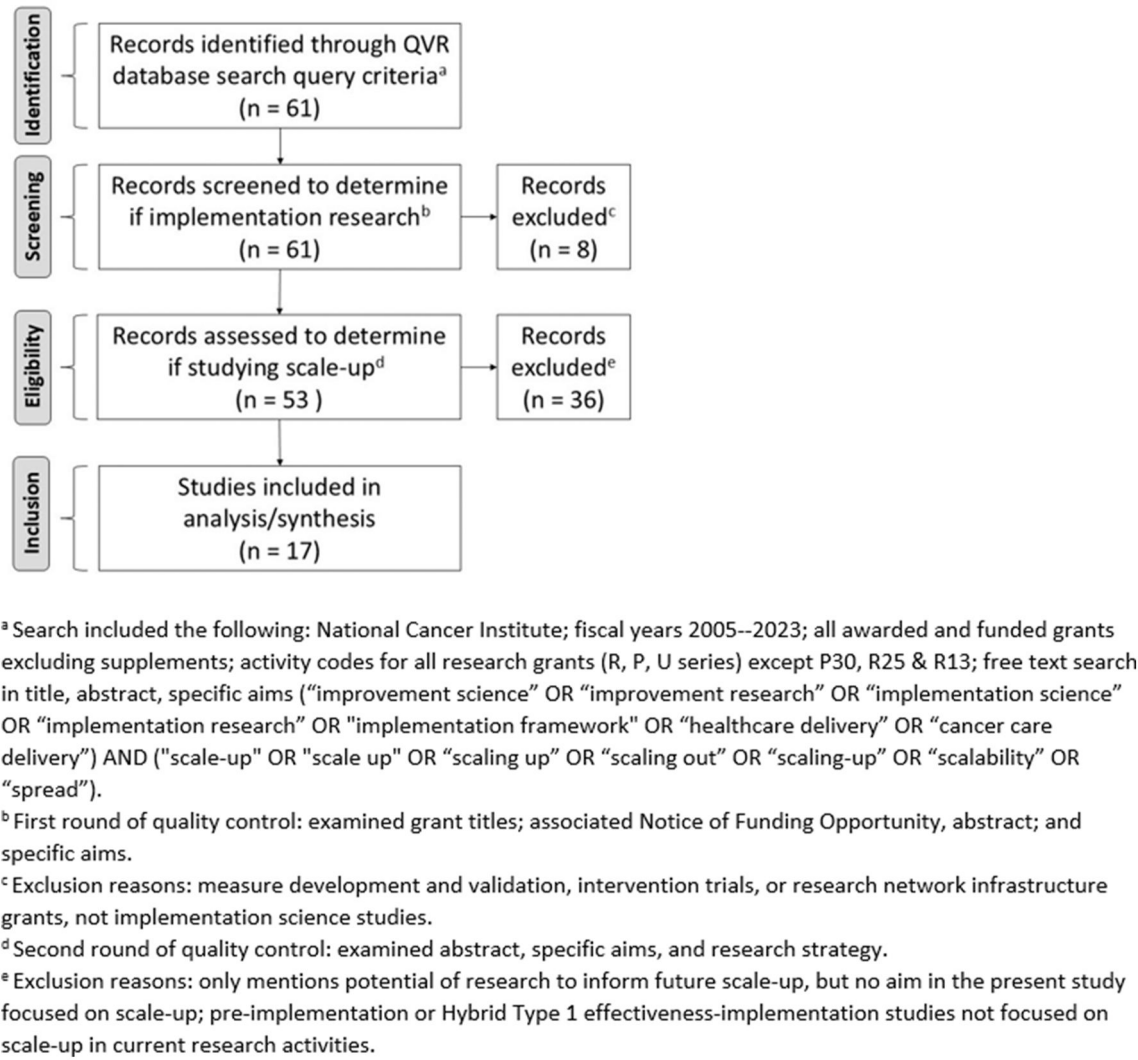
Flow diagram of the identification, screening, eligibility, and inclusion of grants for portfolio analysis.

The search was limited to new and competing continuance applications awarded since 2005, the first year of the NIH-wide Dissemination and Implementation Research in Health funding opportunity (9), with the NCI as the administrative center. The resulting grants were then filtered to capture only P-, U-, and R-series mechanisms, excluding the P30, R13, and R25 mechanisms, which are infrastructure and training grants, and other grant types that are not research studies. The search was conducted in December 2022 and updated in May 2024 using the same procedures both times, to capture any additional grants awarded. We followed the Preferred Reporting Items for Systematic Reviews and Meta-Analyses (PRISMA) Statement guidelines (10) to document the review stages to arrive at a final analytic sample (Figure 1).

We leveraged an internal NIH portfolio analysis software, *iSearch*, which provides access to carefully curated, extensively linked datasets of funded and unfunded grant applications, historical and current, and allows for structured coding by multiple team members. Two research team members examined each grant title, associated Notice of Funding Opportunity [i.e., Dissemination and Implementation Research in Health (e.g., PAR-22-105, PAR-22-106, PAR-22-109), Implementation Science Centers for Cancer Control RFA-CA-19-005 and RFA-CA-19-006], abstract, and specific aims to ensure that the grants identified were truly implementation science. We used the following definition of implementation science as a guide: “the study of methods to promote the adoption and integration of evidence-based practices, interventions, and policies into routine health care and public health settings to improve our impact on population health” (11). Every grant was examined by at least two team members and any discrepancies were resolved by the whole team.

Following quality control to ensure that grants were implementation science, two coders each reviewed the abstracts, specific aims, and research strategies sections of the remaining grants for eligibility to ensure that they were studying scale-up or explicit factors related to scale-up. We used ExpandNet's definition of scaling up as a guide: “deliberate efforts to increase the impact of innovations successfully tested in pilot or experimental projects to benefit more people and to foster policy and program development on a lasting basis” (12). The remaining grants, confirmed to be implementation science studying scale-up, constituted the analytic sample, and the full research strategy section was subsequently reviewed for data extraction by dual coders. All discrepancies were resolved through discussions between the two coders, and as needed, full group discussion.

### Codebook development and coding process

The codebook for this portfolio analysis was developed iteratively based on standard practice guidance from the NIH Office of Portfolio Analysis and an initial review of other NIH- and NCI-specific grant portfolio analysis codebooks and related publications (6, 7, 13, 14). The research team also reviewed the literature on scale-up to develop codes and definitions and held multiple discussions to finalize a codebook that was tailored to the research questions at hand. Variables included in the codebook were study objectives, cancer care continuum, cancer types, content area, study setting, implementer, study design, theory or framework used, and terms used to describe the scale up concept. Detailed categories for each of the codebook variables are described in Appendix A.

Two grants from the analytic sample were selected, one domestic and one international, and coded by all four study team members to pilot the codebook and as a training exercise to ensure consistent understanding of code definitions across the study team. After the two grants were discussed and discrepancies were resolved, minor edits were made to clarify the codes and finalize the codebook (see Appendix A). Two coders each read the full-text specific aims and research strategy for each of the remaining grants and applied corresponding codes from the finalized codebook built into *iSearch*. Discrepancies were resolved through discussion and rereview of the grant content until agreement was reached. In a small number of cases, the discrepancies were brought to the other two research team members for adjudication.

### Data analysis

To characterize the portfolio of grants included in this analysis, descriptive grant metadata were extracted from *iSearch* and downloaded into Microsoft Excel, including administrative data (e.g., year awarded, funding mechanism, study section) and awardee information (e.g., early-stage investigator status). To address the research questions for this study, we extracted data from *iSearch* and downloaded them into Microsoft Excel codes applied to each of the grants. We then calculated frequency and descriptive statistics to characterize the sample of grants according to the codebook variables.

## Results

A total of 61 grants awarded by the NCI were identified through the QVR database search query criteria. After review for inclusion, 17 implementation science grants studying scale-up and awarded by the NCI were identified and coded by paired reviewers. Figure 1 shows the PRISMA flow diagram and the multistep eligibility assessment followed to identify the final sample of grants included in this portfolio analysis.

Table 1 shows the descriptive statistics of the final sample of 17 grants included for full coding and analysis. All grants were awarded between 2016 and 2023, with 47.1% of them awarded in 2022 (*n* = 8). Most of the grants were large research projects using the R01 mechanism (58.8%; *n* = 10), 17.6% were exploratory or developmental research projects (R21; *n* = 3), and 23.5% of the awarded grants were cooperative agreements (U01 = 3 and UM1 = 1).

**Table 1:** Characteristics of included grants (*N* = 17).

Descriptive variable	Total	
	Frequency	Percent
Year awarded		
2023	2	11.8
2022	8	47.1
2021	2	11.8
2018	2	11.8
2017	1	5.9
2016	2	11.8
Funding mechanism^a^		
R01	10	58.8
R21	3	17.6
U01	3	17.6
UM1	1	5.9
Study Section		
DIRH/SIHH^b^	7	41.2
Other	10	58.8

^a^ R01: NIH Research Project Grant Program; R21: NIH Exploratory/Developmental Research Grant Award; U01, UM1: Cooperative Agreements.

^b^ In October 2020, DIRH changed to SIHH; DIRH was the Dissemination and Implementation Research in Health Study Section, SIHH is the Science of Implementation in Health and Healthcare Study Section.

The characteristics of the interventions under study in the grants analyzed are shown in Table 2. Along the cancer control continuum, most grants addressed cancer prevention (64.7%, *n* = 11), followed by screening (41.2%, *n* = 7), treatment (11.8%, *n* = 2), diagnosis (11.8%, *n* = 2), and/or survivorship (11.8%, *n* = 2). Tobacco control (23.5%, *n* = 4) was the most commonly studied area after cancer screening. Cervical (40.2%, *n* = 6) and lung (20.0%, *n* = 3) cancers were the most studied.

**Table 2:** Cancer-related characteristics of the interventions under study (*N* = 17).

Characteristics	Total	
	Frequency	Percent
Cancer Control Continuum Area^a^		
Prevention	11	64.7
Screening	7	41.2
Treatment	2	11.8
Diagnosis	2	11.8
Survivorship	2	11.8
Cancer Content Area^a^		
Screening	7	41.2
Tobacco control	4	23.5
Vaccine uptake	3	17.6
Diet/Nutrition	2	11.8
Symptom management	2	11.8
Patient navigation	1	5.9
Sun safety	1	5.9
Physical activity	1	5.9
Skin self-examination	1	5.9
Quality of life	1	5.3
HIV treatment adherence	1	5.3
Cancer Type		
Cervical	6	40.0
Lung	3	20.0
Skin	2	13.3
Colorectal	2	13.3
HPV-related	1	6.7
Head and neck	2	6.7
HIV-related	1	6.7
Liver	1	6.7
Breast	1	6.7
Non-specified	2	13.3

^a^ Responses not mutually exclusive.

Table 3 lists the implementation-related characteristics of the studies included in the analysis, with 64.7% (*n* = 11) being delivered in a healthcare setting. Most grants were delivered by healthcare providers (35.3%, *n* = 6), followed by peer/lay healthcare professionals (29.4%, *n* = 5), public health officials (15.8%, *n* = 4), clinic support staff (17.6%, *n* = 3), and electronic health or medical records (10.5%, *n* = 2) or mobile devices (10.5%, *n* = 2). Most grants included in the analysis were based in the United States (64.7%, *n* = 11), with the remainder studying populations in LMICs, including Nigeria, India, Rwanda, Zambia, and Peru (35.3%, *n* = 6).

**Table 3:** Characteristics of included studies (*N* = 19).

Characteristics	Total	
	Frequency	Percent
Type of Organization, Delivery System or Setting^a^		
Healthcare	11	64.7
Health department	2	11.8
Community-based organization	2	11.8
School	2	11.8
Workplace	2	11.8
Means of Intervention Delivery^a^		
Healthcare provider	6	35.3
Peer/Lay healthcare professional	5	29.4
Public health official	4	15.8
Clinic support staff	3	17.6
Electronic health or medical record	2	10.5
Mobile device	2	10.5
Administrators/Policymakers	1	5.9
Employer	1	5.9
Other technology	1	5.9
School personnel	1	5.9
Other delivery mode	1	5.9
Not applicable	2	11.8
Low- and middle income country^b^		
No	11	64.7
Yes	6	35.3
Number of Sites Studied for Scale-up		
6–10	3	17.6
11–40	7	41.2
>40	5	29.4
Not applicable	2	11.8
Study Objectives^a^		
Examine scale-up related factors (barriers/facilitators, context)	11	64.7
Assess costs and benefits of scaling	9	52.9
Evaluate an implementation strategy to scale	7	41.2
Measure scale-up of the intervention	3	17.6
Other	2	11.8
Study Design^a^		
Mixed Methods	12	70.6
Experimental	10	58.8
Observational	5	29.4
Qualitative	4	21.1
Quasi-experimental	2	10.5
Theory/Model/Framework^a^		
Consolidated Framework for Implementation Research (CFIR)	9	52.9
Practical Robust Implementation and Sustainability Model (PRISM) or Reach Effectiveness Adoption Implementation Maintenance (RE-AIM) (29)	7	41.2
Proctor's Implementation Outcomes Framework (30)	2	11.8
Capability, Opportunity, Motivation, Behavior (COM-B) model (31) or Theoretical Domains Framework (TDF) (32)	2	11.8
Dynamic Sustainability Framework (21)	2	11.8
CDC's Policy Analytical Framework (20)	1	5.9
Diffusion of Innovations (DOI) (33)	1	5.9
Exploration Preparation Implementation Sustainment (EPIS) (34)	1	5.9
Health Promotion Research Center Dissemination and Implementation Framework (19)	1	5.9
Model for Adaptation Design and Impact (MADI) (35)	1	5.9
Socioecological Model (36)	1	5.3
Terms Used^a^		
Scale-up	16	94.1
Scalability	8	47.1
Other	1	5.9

^a^ Responses not mutually exclusive.

^b^ Classification per Wellcome Trust: https://wellcome.org/grant-funding/guidance/low-and-middle-income-countries

The scope of scale-up varied widely across grants in terms of the number of sites included, with a mean of 53 sites, ranging from six to two hundred across seventeen studies. Most studies (64.7%, *n* = 11) aimed to examine factors (barriers and facilitators) related to the scale-up of an intervention. For example, examining themes related to the outer setting and individual or intervention characteristics that may impede or facilitate scalability of the intervention or implementation strategy. To examine these factors, most studies have proposed conducting postimplementation process evaluations to identify stakeholders' viewpoints on barriers and enablers to scale the intervention to other clinics or across the country of study. Nine grants (52.9%) assessed the costs and benefits of the intervention. For example, one grant proposed conducting economic analyses using a micro-costing approach to model and compare the cost-effectiveness of two sun safety workplace interventions and determine the costs and implementation rates needed to scale-up and achieve the desired reach. Several grants (41.2%, *n* = 7) proposed to develop and evaluate an implementation strategy. For example, one study proposed developing and evaluating the effectiveness of a scale-up strategy to analyze human papillomavirus (HPV) test adoption, screening coverage, and completion of care in multiple health systems using a mixed-method design. Few studies (17.6%, *n* = 3) proposed to measure scale-up, often through process measures (e.g., organizational changes in policy and education). Finally, two studies had additional aims (other category) to optimize intervention components that are cost-effective and scalable as well as evaluate the potential for scale-up of an implementation toolkit.

Most grants proposed mixed methods study designs (70.6%, *n* = 12) and involved engaging stakeholders at multiple levels. Additionally, 58.8% (*n* = 10) of the studies had an experimental design. For example, one grant proposed a hybrid type III design (15) to conduct a randomized controlled trial comparing the effects of two training strategies. The authors of this study justified the selection of a hybrid type III design, as it facilitates the identification of an effective and sustainable delivery method to scale up the intervention beyond the local setting. Grants with study designs including some observational component (36.8%, *n* = 7) proposed, for example, site observations to identify available resources and the capacity to scale the intervention. Three studies (17.6%) employed qualitative methods only.

Nine grants coded for this portfolio analysis (52.9%) were guided by the Consolidated Framework for Implementation Research (CFIR), whereas 41.2% (*n* = 7) used the Practical Robust Implementation and Sustainability Model (PRISM) (16) or the Reach, Effectiveness, Adoption, Implementation, and Maintenance (RE-AIM) framework (17, 18). For example, one grant adapted the RE-AIM framework to incorporate costs and resources for intervention delivery as important components of the proposed scale-up study. Examples of other theories, models, or frameworks used to study scale-up in these grants included the Health Promotion Research Center Dissemination and Implementation Framework (19), CDC's Policy Analytical Framework (20), and the Dynamic Sustainability Framework (21). Finally, we coded for the terms used: 94.1% (*n* = 16) of grants used scale-up, 47.1% (*n* = 8) scalability, and one grant used the term penetration.

## Discussion

Reviewing the NCI portfolio of implementation science grants focused on scale-up reveals relatively few grants in this space, but the number has been steadily increasing since 2016, peaking in 2022. There is a heavy focus on prevention and early detection in the grant portfolio, which aligns with the National Cancer Institute goals to prevent most cancers before they start and ensure the best outcomes for every person diagnosed with cancer. However, there is a gap in scaling-up EBIs following a cancer diagnosis in the treatment and survivorship phases that investigators could address to further advance national goals. Expanding the scope of implementation studies beyond small-scale, few settings to examine scaling implementation to multiple sites and settings can improve the reach of EBIs by informing scale up strategies.

Some types of cancer screening, such as breast and cervical cancer screening, along with tobacco control, are among the longest standing EBIs available for the prevention and control of cancer (22), so it is unsurprising that they might be most primed for scale-up research and most heavily represented in the portfolio. Similarly, the most common cancers addressed by grants in this portfolio were cervical and lung. This is also unsurprising given the increasing focus in recent years on the elimination of cervical cancer with Pap testing, HPV testing, and HPV vaccination (23). Similarly, lung cancer, the predominant cause of cancer mortality globally, also has prevention and early detection EBIs available now in tobacco prevention and cessation as well as low-dose CT lung cancer screening (24, 25).

In an effort to stimulate more scale-up focused implementation science, NCIs recently published linked cooperative agreement funding opportunities (UG3/UH3) for the Scaling-Up and Maintaining Evidence-Based Interventions to Maximize Impact on Cancer (SUMMIT) initiative focused on lung cancer screening and tobacco use treatment for cancer survivors (RFA-CA-25-009 and RFA-CA-25-010) (26, 27). Up to three clinical trials for each topic will test implementation strategies to equitably and effectively scale up and sustain the delivery of EBIs to a large number (at least 60 sites each) of diverse clinical care delivery settings, with an emphasis on populations experiencing health inequities. The coordination of strategies and common data elements across the initiative is intended to enable greater discovery of scale-up and sustainability-specific implementation strategies, generating useful evidence for practice and policy.

Healthcare has been the primary setting for the delivery of cancer-related EBIs and implementation science and was accordingly well represented in this portfolio. However, investigators may wish to explore opportunities to expand the delivery of cancer-related EBIs to nontraditional community settings via various technologies to reduce the burden on strained health systems and providers and expand the EBI reach to populations who may not be connected with the healthcare system. Milat, Bauman, and Redman (2015) previously identified eight frameworks that could be used to help scale EBIs into practice and policy, and this portfolio analysis confirmed that a number of theories, models, and frameworks from implementation science, public health, and policy fields can be leveraged in scale-up research (28).

In terms of scale-up related features of grants in the portfolio, there was a wide range of sites included for scale-up, from as few as six to as many as two hundred and only five studies looked at scaling an EBI to more than forty sites. If we are to achieve national cancer goals at a societal level, it will be necessary to continue to push for scaling EBIs to a greater number of sites and individuals. Additionally, while studying factors related to scale-up is critical and was included in most grants, the portfolio analysis revealed gaps in evaluating strategies for scale-up, which may differ from implementation strategies for implementation at a single site in terms of burden, cost, reach, acceptability, etc., or in different political or healthcare contexts globally. Indeed, a systematic review by Bulthuis et al. (2019) reported factors influencing the scale-up of public health EBIs in LMICs, examining “vertical scale-up for sustainability” and highlighting factors that influence changes in structure, practice, and culture (3). There was also a gap in terms of measuring scale-up, as only three grants did so. This is consistent with a prior review of scale-up research not limited to cancer, where outcomes measured tend to be at the patient or provider level rather than specific to scale-up (2). Measuring scale-up directly can inform system-level strategies to improve implementation at scale across a range of heterogeneous settings.

Limitations of this analysis include the possibility that we may have missed relevant grants. However, we tried to perform as exhaustive a search as possible, using a range of search terms based on terms used in previously published reviews. Further, the generalizability of our findings may be limited for other funding entities since we focused on NCI-funded grants only.

## Conclusion

Although NCI has funded a limited number of grants focused on scale-up, opportunities are growing, as demonstrated by the recent issuance of SUMMIT. This analysis helps identify the current scope of the NCI portfolio and enables exploration of gaps and opportunities for future research on scale-up across the cancer continuum.

## Data Availability

The datasets presented in this article are not readily available because Grant applications are confidential and accessible only to U.S. NIH staff. Requests to access the datasets that are publicly available should be directed to Gila.Neta@nih.gov.

## Appendix A. Codebook variables, questions, and codes

**Table:**

Variable	Question and Codes
Objectives	What is/are the overall study objective(s)? Select all that apply. (Variable: Objectives) a) Examine factors (barriers/facilitators, context) related to scale-up b) Evaluate the impact of an implementation strategy on the scale-up of evidence-based intervention, program, or policy c) Measure the scale-up of EBPs, strategies, practices, or health outcomes (without testing an implementation strategy) d) Assess costs and benefits of the scaled-up delivery of the evidence-based intervention, program, or policy. e) Methodological (e.g., developing measure, testing TMF) f) Other, please specify
Cancer continuum	Where is the study focused along the cancer control continuum? *Select all that apply.* a) Prevention b) Screening c) Diagnosis d) Treatment e) Survivorship f) End of life (hospice)
Cancer type(s)	What cancer type is the focus of the study? *Select all that apply.* a) Anal cancer b) Breast cancer c) Cervical cancer d) Colorectal cancer e) Liver cancer f) Lung cancer g) Prostate cancer h) Skin cancer i) HIV related cancers j) HPV related cancers k) Other, please specify l) Not specified
Content	What cancer content area does the study focus on? What is the focus of the intervention? *Select all that apply unless encompassed by comprehensive content area (e.g., survivorship care).* a) Comorbidity disease management (e.g., cardiovascular disease; obesity) b) COVID-19 c) Diet/Nutrition d) Fertility e) Financial hardship f) Genetic services (testing, screening and counseling) g) Patient navigation h) Physical activity i) Psychological well-being (e.g., depression, anxiety, quality of life) j) Risk assessment (e.g., hereditary cancer, cervical cancer, risk-stratified screening, risk prediction models, polygenic risk scores) k) Screening l) Shared decision making m) Sun Safety n) Tobacco control o) Social determinants of health (e.g., supportive housing, social services) p) Survivorship care (e.g., treatment decision making, patient-caregiver social support) q) Symptom management (e.g., pain) r) Vaccine uptake s) Other, please specify t) Not specified
Country	What is the country where scale-up is being studied? *Open-ended response*
LMIC	Is the country a low- or middle-income country as classified by the Organisation for Economic Co-operation and Development (OECD)? https://www.oecd.org/dac/financing-sustainable-development/development-finance-standards/daclist.htm a) Yes b) No
Setting	In what type of organization, delivery system, or setting does the study take place? Where/how is the intervention delivered/will be delivered? Or through what settings? *Select all that apply. Not recruitment setting, intervention delivery setting.* a) Community-based organization (e.g., social service organizations, churches) b) Healthcare settings (e.g., integrated delivery system, community health centers, FQHCs academic medical center) c) Recreation area (e.g., park, pool, beach) d) School (e.g., elementary/junior/high school, college/university excluding academic medical centers) e) Health department (e.g., state, local) f) Workplace setting (e.g., businesses, corporations) g) Other, please specify h) Not specified
Implementer/Deliverer	By whom is the intervention being delivered? *Select all that apply.* a) Peer/Lay healthcare professionals (e.g., peer educators, patient navigators, community health workers) b) Healthcare Provider (e.g., nurses, physicians, specialists) c) Clinic support staff (e.g., medical assistant) d) Public health official (e.g., state or local health department staff) e) School personnel f) Not stated g) Other, please specify
Design	What type of study design is used? *Select all that apply.* a) Experimental (RCT, pragmatic RCT, cluster/group RCT) b) Mixed methods (i.e., collection and integration of qualitative and quantitative data - e.g., sequential exploratory design) c) Observational (e.g., prospective, retrospective) d) Qualitative (e.g., focus groups, semi-structured interviews) e) Quasi-experimental (e.g., regression discontinuity, interrupted time series) f) Measurement or algorithm development or validation g) Systems science (e.g., simulation modeling, social network analysis)
TMFs	List implementation science theories, models, or frameworks used. *Select all that apply.* a) CFIR b) RE-AIM/PRISM c) EPIS d) PARIHS/I-PARIHS e) Other, please specify
Terms	Which terms were used to describe scale-up? *Select all that apply.* a) Scale-up b) Scaling out c) Up-scaling d) Scalability e) Spread f) Other, please specify
